# The Atherogenic Index of Plasma is a Predictor for Chronic Total Occlusion and Coronary Collateral Circulation Formation in CTOs Patients

**DOI:** 10.31083/j.rcm2410305

**Published:** 2023-10-23

**Authors:** Ya Li, Yujia Feng, Ya Zhong, Shu Li, Jiesheng Lin, Peng Fang, Jing Wan, Min Zhao

**Affiliations:** ^1^Department of Cardiology, Zhongnan Hospital of Wuhan University, 430071 Wuhan, Hubei, China; ^2^Department of Geratology, Zhongnan Hospital of Wuhan University, 430071 Wuhan, Hubei, China; ^3^Department of Intensive Care Unit, Zhongnan Hospital of Wuhan University, 430071 Wuhan, Hubei, China; ^4^Institute of Epidemiology, Helmholtz Zentrum München, German Research Center for Environmental Health, 85764 Munich, Germany; ^5^Department of Cardiology, Huangshi 5th Hospital, 435005 Huangshi, Hubei, China; ^6^Demonstration Center for Experimental Basic Medicine Education, Taikang Medical School (School of Basic Medical Sciences), Wuhan University, 430071 Wuhan, Hubei, China

**Keywords:** atherogenic index of plasma, coronary collateral circulation, chronic total occlusive disease, coronary angiography, diagnosis

## Abstract

**Background:**

The atherogenic index of plasma (AIP), determined by the logarithmic transformation of the ratio of triglyceride (TG) and high-density lipoprotein cholesterol (HDL-C), was found to be a marker of cardiovascular disease. We sought to investigate the correlation between the atherogenic AIP and coronary collateral circulation (CCC) formation in chronic total occlusive (CTOs) patients.

**Methods:**

This retrospective cohort study included 665 non-CTOs and 345 CTOs patients. CTOs were divided into 206 CCC poor formation patients and 139 CCC good formation patients according to the Cohen-Rentrop grade. Spearman correlation analysis was carried out to obtain the relationship between AIP and the Rentrop grade. We used multivariate logistic regression analysis to assess CTOs and CCC poor formation risk factors. Receiver operating characteristic (ROC) curves were used to determine the optimal threshold for AIP to predict CTOs and CCC poor formation. The predicted increment of AIP on CTOs and CCC poor formation was evaluated by calculating the Net Reclassification Index (NRI) and the Integrated Discriminant Index (IDI).

**Results:**

AIP in CTOs was significantly elevated compared to non-CTOs patients [(1.55 (1.02, 2.59)) *vs* (1.26 (0.82, 1.90)), *p* < 0.001] AIP in the CCC poor formation group was significantly higher than that in the CCC good formation group [(1.73 (1.12, 2.90)) *vs* (1.37 (0.84, 2.13)), *p* = 0.002]. There was a negative correlation between AIP and the Rentrop grade (r = –0.145, *p* = 0.007). The results of multivariate logistic regression revealed that AIP was an independent predictor of CTOs (OR = 4.371, 95% CI: 2.436–7.844, *p* < 0.001) and CCC poor formation (OR = 3.749, 95% CI: 1.628–8.635, *p* = 0.002). In the ROC analysis, the area under the curve of AIP for identifying CTOs and CCC poor formation was 0.596 (OR = 3.680, 95% CI: 1.490–9.090, *p* = 0.005) and 0.597 (95% CI: 0.535–0.658, *p* = 0.002), respectively.

**Conclusions:**

Contrary to previous research, we found that AIP is a moderate but not powerful indicator for detecting both CTO patients and poor CCC formation.

## 1. Introduction

Chronic total occlusions (CTOs) are a common finding in patients with coronary artery disease (CAD), with a reported prevalence of approximately one-third in this patient population. This is a major global health concern in the field of cardiovascular medicine [1]. CTOs are frequently seen during coronary angiography and involve coronary artery complete occlusion for more than 3 months. Studies have indicated that the presence of CTOs is associated with a higher incidence of major adverse cardiovascular events (MACEs). This highlights the importance of identifying and treating CTOs in patients with CAD to reduce the risk of adverse cardiovascular outcomes [2]. Coronary collateral circulation (CCC) is an arterial anastomosis network that forms a natural bypass through arteriogenesis or lumen expansion when there is insufficient blood flow in the distal myocardium when coronary artery occlusion occurs [3]. A reduction in mortality and an improvement in cardiac function is associated with the presence of well-formed collaterals, which has cardioprotective effects on ischemic myocardium [4, 5]. Therefore, it is necessary to predict and evaluate the formation of CCC in clinical practice. However, the current methods to evaluate CCC such as the Collateral Flow Index (CFI) and intracoronary electrocardiogram are relatively complex and expensive. Therefore, there is a need for a simple and cost-effective method to effectively predict or evaluate the formation of CCC in CTO patients.

The atherogenic index of plasma (AIP), determined by the logarithmic transformation of the ratio of triglyceride (TG) and high-density lipoprotein cholesterol (HDL-C), has been identified as a potential marker of cardiovascular disease. Studies have shown that AIP is associated with both the onset of CAD and the severity of coronary syndromes [6]. According to a recent study, there is a correlation between AIP levels and the severity of CTO. This suggests that AIP may be a useful biomarker for predicting the occurrence of CTO in patients with CAD [2]. Previous research has suggested that AIP levels may be an independent predictor of the complexity of CTO. This highlights the potential value of AIP as a biomarker for predicting not only the occurrence of CTO, but also their severity and complexity. In addition to the Japanese Multicenter CTO Registry (J-CTO) score, AIP may be associated with the number and length of stents used after successful revascularization [7]. However, the relationship between AIP and CTO and CCC formation has not been well studied. Therefore, we investigated the relationship between AIP and CTO as well as CCC formation to determine the effectiveness of AIP in the prediction and prognostic assessment of CTO patients in clinical practice.

## 2. Methods

### 2.1 Study Population

Between January 2013 and September 2018, patients who underwent coronary angiography in the Department of Cardiology, Zhongnan Hospital of Wuhan University in whom the coronary angiography showed a 90% stenosis in at least one branch of the three main coronary arteries (left anterior descending artery (LAD), left circumflex artery (LCA), and right coronary artery (RCA)) were consecutively enrolled [8]. CTO was defined as complete coronary artery occlusion due to thrombosis or atherosclerosis, with a duration of occlusion that lasted more than three months.

Exclusion criteria: Previous cardiovascular disease within three months (acute myocardial infarction, coronary stent placement or coronary artery bypass graft, heart failure), cardiomyopathy, congenital coronary arterial malformation or other severe diseases (liver and kidney failure, thyroid dysfunction) and patients taking lipid-lowering drugs for more than three months.

We followed the 2007 STROBE statement for reporting of observational studies [9].

### 2.2 Coronary Collateral Circulation Assessment and Grouping

Coronary angiography was performed by two interventional experts using the Judkin method by the radial or femoral approach. The Cohen-Rentrop criterion was used to evaluate CCC formation [10]: Grade 0, without any filling of the collateral arteries; Grade 1, the side branches of the occluded artery can be filled but the contrast agent cannot reach the epicardial vessel; Grade 2, partial filling of epicardial vessels; and Grade 3, epicardial vessels are completely filled. Based on the angiography results, the participants were divided into a CTOs group and a non-CTOs group. In addition, patients with CTO were separated into the CCC poor formation group (Rentrop Grade 0–1) and the CCC good formation group (Rentrop Grade 2–3).

### 2.3 Laboratory Measurements

Venous blood was obtained from patients who had fasted for more than 10 hours. Blood lipids were measured using an automated biochemical analyzer (Beckman Coulter, AU5800, Brea, CA, USA) and an enzymatic reaction method. TG/HDL was calculated as the ratio of serum TG (mmol/L) to serum HDL-C (mmol/L), and AIP was defined as the logarithm of the ratio of TG/HDL-C.

### 2.4 Statistical Analysis

The Kolmogorov–Smirnov test was used to test the normality of data distribution. Continuous data are shown as the mean ± SD for normal distribution and medians (interquartile ranges) for data with a non-normal distribution. Student’s *t*-test and Mann–Whitney U-test were used to identify differences between the two groups, respectively. Categorical variables were expressed as percentages and compared using the χ^2^-test. Categories of the Rentrop grades were compared using one-way analysis of variance (ANOVA). We used Spearman’s correlation analysis to describe the correlation between AIP and the Rentrop grade. Multivariate binary logistic regressions were used to estimate the association of AIP with prevalent CTOs (yes or no) and CCC formation (good or poor). Before performing multivariate binary logistic regression analysis, collinearity diagnosis was performed on the included variables. Variables with tolerance <0.1 or variance inflation factor (VIF) >10 were excluded. Receiver operating characteristic (ROC) curves were used to assess the predictive ability of AIP for CTOs and CCC poor formation. The statistical analyses were performed using IBM SPSS 23.0 software (IBM Corp, Armonk, NY, USA). In addition, we also used R software version 4.0.2 (R development Core Team, Vienna, Austria, https://www.R-project.org) to calculate the net reclassification index (NRI) and the integrated discrimination index (IDI) to better evaluate the predicted incremental value of AIP. In accordance with previous studies, we considered setting the threshold as B/2, B, 2B according to the incidence of CTO and poor CCC formation (B stands for incidence) [11, 12, 13, 14, 15, 16, 17, 18, 19], and divided patients into four risk groups. The results are represented by 95% confidence intervals (CI). A *p* < 0.05 means represented statistical significance.

## 3. Results

### 3.1 Basic Clinical Information and Characteristics of Patients

**Fig. 1.**
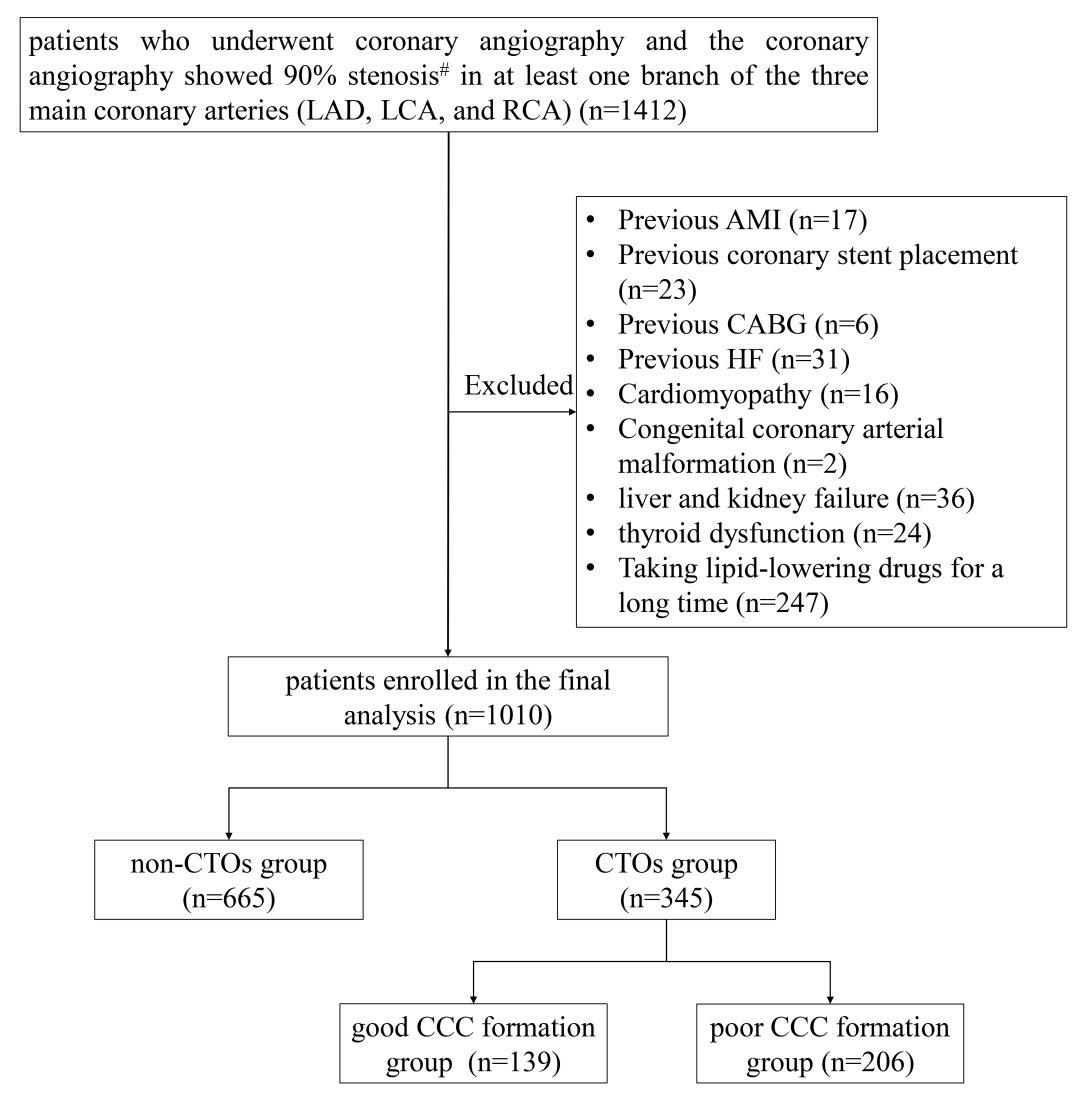
**Flowchart of the cohort patients.** LAD, left anterior descending artery; LCA, left circumflex artery; RCA, right coronary artery; AMI, acute myocardial infarction; CABG, coronary artery bypass graft; HF, heart failure; CTO, chronic total occlusive; CCC, coronary collateral circulation. ^#^Coronary artery stenosis is assessed visually by coronary angiography.

A total of 1412 patients who underwent coronary angiography in the Department of Cardiology, Zhongnan Hospital of Wuhan University were consecutively enrolled (Fig. 1). 402 patients who met the exclusion criteria were excluded. In total, 1010 patients were enrolled in the study, of whom 665 were non-CTO patients and 345 were CTO patients. Among the CTO patients, 139 patients were assigned to the good CCC formation group and 206 patients were assigned to the poor CCC formation group. Table 1 shows that 665 non-CTOs patients and 345 CTOs patients were included in this study. Patients in the CTOs group were older, more likely to be male and to have a history of smoking, diabetes, and hypertension, and a higher Lp(a) and AIP, but lower HDL levels.

**Table 1.** **Basic clinical characteristics of the study participants**.

Variables	non-CTOs group (n = 665)	CTOs group (n = 345)	*p* value
Age	58.51 ± 11.00	62.20 ± 11.61	< ${\mathrm{0.001}}^{1}$
Gender (men (%))	353 (55.2)	286 (82.9)	< ${\mathrm{0.001}}^{1}$
Smoking	138 (20.8)	165 (47.8)	< ${\mathrm{0.001}}^{1}$
Diabetes	80 (12.0)	99 (28.7)	< ${\mathrm{0.001}}^{1}$
Hypertension	295 (58.2)	212 (61.4)	< ${\mathrm{0.001}}^{1}$
TC (mmol/L)	4.46 ± 1.06	4.87 ± 1.04	0.838
TG (mmoL/L)	1.45 (1.01, 2.07)	1.51 (1.06, 2.24)	0.081
HDL (mmol/L)	1.81 ± 0.30	1.01 ± 0.27	< ${\mathrm{0.001}}^{1}$
LDL (mmol/L)	2.68 ± 0.86	2.79 ± 0.80	0.052
LP(a) (mg/L)	109.95 (53.53, 210.63)	127.70 (65.40, 243.65)	${\mathrm{0.011}}^{2}$
AIP	1.26 (0.82, 1.90)	1.55 (1.02, 2.59)	< ${\mathrm{0.001}}^{1}$

CTOs, chronic total occlusions; TC, total cholesterol; TG, triglyceride; HDL, high-density lipoprotein; LDL, low-density lipoprotein; LP(a), Lipoprotein(a); AIP, atherogenic index of plasma. Note: ^1^*p* < 0.01, ^2^*p* < 0.05.

### 3.2 Clinical Baseline and Characteristics of CTOs Patients

According to the Cohen-Rentrop criteria and the results of the CCC formation evaluation, CTOs patients were divided into good and poor CCC groups, and AIP was compared between these two groups. Table 2 showed that the CCC poor formation group had a higher AIP ((0.14 ± 0.30) *vs* (0.26 ± 0.31)) level. The proportion of multivessel lesions in the CCC good formation group was higher, suggesting that multivessel lesions may contribute to the formation of CCC.

**Table 2.** **Basic clinical characteristics of CTOs patients**.

Variables		Good CCC formation group (n = 139)	Poor CCC formation group (n = 206)	*p* value
Age		63.30 ± 10.80	61.46 ± 12.10	< ${\mathrm{0.001}}^{1}$
Gender [man (%)]		120 (42.0)	166 (58.0)	0.190
Smoking		68 (41.2)	97 (58.8)	0.743
Diabetes		38 (38.4)	61 (61.6)	0.716
Hypertension		92 (43.4)	120 (56.6)	0.144
TC (mmol/L)		4.31 ± 0.90	4.59 ± 1.12	${\mathrm{0.010}}^{2}$
TG (mmoL/L)		1.45 (1.01, 2.07)	1.51 (1.06, 2.24)	0.081
HDL (mmol/L)		1.06 ± 0.30	0.98 ± 0.24	${\mathrm{0.010}}^{1}$
LDL (mmol/L)		2.69 ± 0.71	2.86 ± 0.85	${\mathrm{0.048}}^{2}$
LP(a) (mg/L)		109.95 (53.53, 210.63)	127.70 (65.40, 243.65)	${\mathrm{0.011}}^{2}$
AIP		1.37 (0.84, 2.13)	1.73 (1.12, 2.90)	${\mathrm{0.002}}^{1}$
Occlusive vessel (%)				
	Multiple vessels lesions	31 (63.3)	18 (36.7)	${\mathrm{0.001}}^{1}$
	LAD	37 (37.4)	62 (62.6)	0.544
	LCX	19 (27.1)	51 (72.9)	${\mathrm{0.014}}^{2}$
	RCA	52 (40.9)	75 (59.1)	0.909

CTOs, chronic total occlusions; CCC, coronary collateral circulation; TC, total cholesterol; TG, triglyceride; HDL, high-density lipoprotein; LDL, low-density lipoprotein; LP(a), Lipoprotein(a); LAD, left anterior descending artery; AIP, atherogenic index of plasma; RCA, right coronary artery; LCX, left circumflex artery. Note: ^1^*p* < 0.01, ^2^*p* < 0.05.

### 3.3 Correlation between AIP and Rentrop Grade

Spearman’s correlation analysis was carried out to obtain the relationship between AIP and the Rentrop grade. The results showed that AIP (r = –0.145, *p* = 0.007) was negatively correlated with Rentrop grade. The AIP in Rentrop 3 (1.22 (0.8, 1.94)) and Rentrop 2 (1.53 (0.89, 2.36)) were significantly lower than Rentrop 0 (1.65 (1.12, 2.9)) and Rentrop 1 (1.97 (1.14, 2.85)). The AIP between Rentrop 3 and Rentrop 2 (*p* = 0.269), Rentrop 0 and Rentrop 1 (*p* = 0.351) showed no significant differences.

### 3.4 Analysis of Risk Factors for CTOs and CCC Formation

The CTOs (yes or no) and CCC formation (good or poor) were used as the dependent variables. Variables with a univariate relationship with outcome and clinical relevance were included in multivariate models to reveal potential risk factors for CTO and CCC. Since the tolerance of TG and HDL-C were both <0.1, these two variables were not included in the final multivariate binary logistic analysis. After adjusting traditional cardiovascular risk factors (age, gender, smoking, diabetes, and hypertension) and plasma lipid parameters (TC, LDL-C, Lp(a)), AIP was significantly positively associated with a higher prevalence of CTOs (OR = 4.371, 95% CI: 2.436–7.844) (Table 3). In the comparison between the good CCC formation group and the poor CCC formation group, after adjusting traditional cardiovascular risk factors (age, gender, smoking, diabetes, and hypertension), plasma lipid parameters (TC, LDL-C, Lp(a)) and multivessel disease, AIP was significantly positively associated with CCC poor formation (OR = 3.680, 95% CI: 1.490–9.090) (Table 4). The higher the AIP, the more patients tended to have CTOs and poor CCC formation.

**Table 3.** **Logistic regression analysis of CTOs risk factors**.

Variables	B	Wald	OR	95% CI	*p* value
Age	0.052	44.151	1.053	1.037–1.069	<0.001
Sex	–1.374	46.014	0.253	0.170–1.069	<0.001
Smoking	0.849	22.374	2.337	1.644–3.323	<0.001
Diabetes	0.875	19.100	2.399	1.620–3.551	<0.001
Hypertension	0.481	8.990	1.618	1.181–2.217	0.003
TC (mmol/L)	–0.369	5.547	0.692	0.509–0.940	0.019
LDL (mmol/L)	0.557	8.627	1.746	1.204–2.532	0.003
Lp(a) (mg/L)	0.001	7.420	1.001	1.000–1.002	0.006
AIP	1.475	24.455	4.371	2.436–7.844	<0.001

OR, odds ratio; CI, confidence interval; TC, total cholesterol; CTOs, chronic total occlusions; LDL, low-density lipoprotein; LP(a), Lipoprotein(a); AIP, atherogenic index of plasma.

**Table 4.** **Logistic regression analysis of CCC formation risk factors**.

Variables	B	Wald	OR	95% CI	*p* value
Age	0.000	0.001	1.000	0.978–1.023	0.970
Sex	0.461	1.764	1.585	0.803–3.130	0.184
Smoking	–0.074	0.082	0.928	0.559–1.543	0.775
Diabetes	0.032	0.015	1.033	0.615–1.734	0.903
Hypertension	–0.411	2.767	0.663	0.408–1.076	0.096
Multivessel	–1.183	12.519	0.306	0.159–0.590	0.001
TC (mmol/L)	–0.005	0.000	0.995	0.621–1.595	0.984
LDL (mmol/L)	0.330	1.134	1.391	0.758–2.556	0.287
Lp(a) (mg/L)	–0.001	2.610	0.999	0.998–1.000	0.106
AIP	1.303	7.978	3.680	1.490–9.090	0.005

OR, odds ratio; CI, confidence interval; TC, total cholesterol; LDL, low-density lipoprotein; LP(a), Lipoprotein(a); CCC, coronary collateral circulation; AIP, atherogenic index of plasma.

### 3.5 ROC Curve Analysis of the AIP in CTOs and CCC Poor Formation

**Fig. 2.**
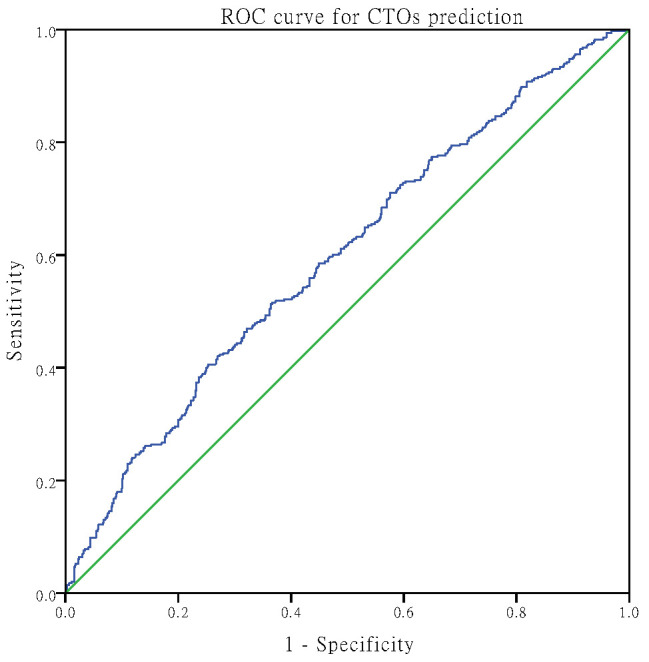
**AIP prediction of CTOs with ROC curves.** AIP, atherogenic index of plasma; CTOs, chronic total occlusions; ROC, receiver operating characteristic.

**Fig. 3.**
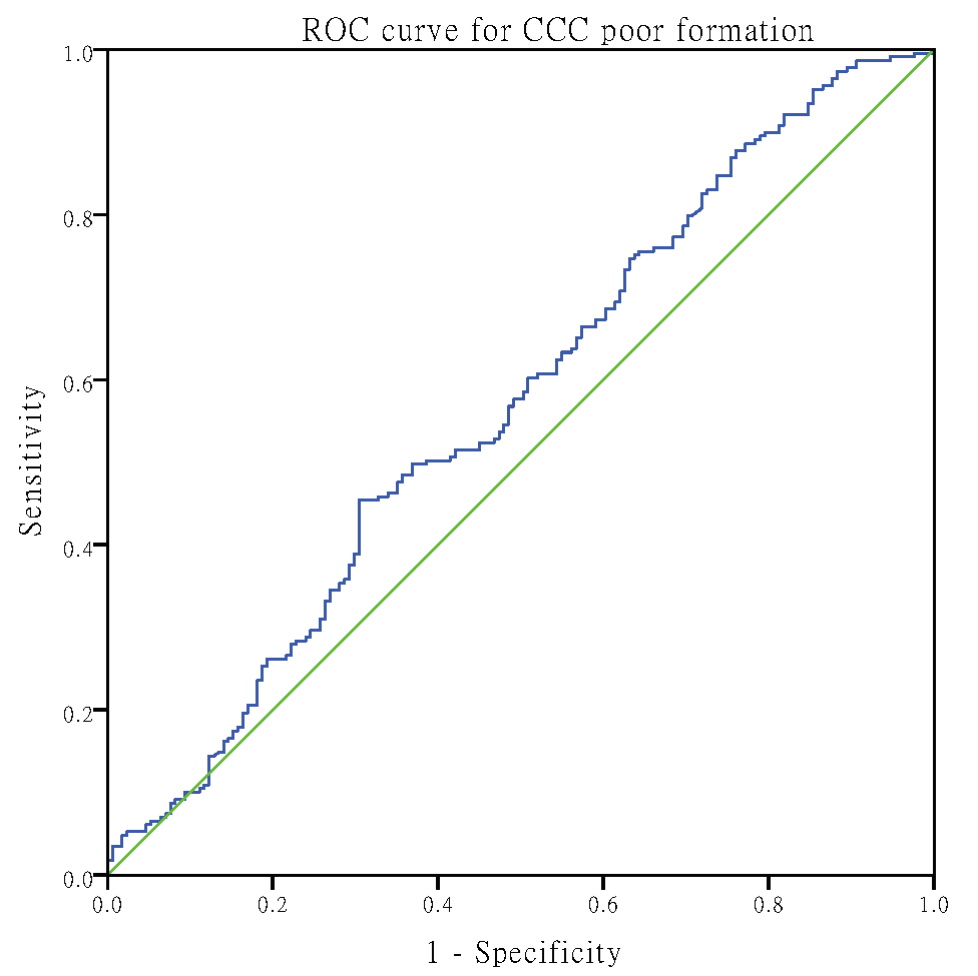
**AIP prediction of CCC poor formation with ROC curves.** CCC, coronary collateral circulation; ROC, receiver operating characteristic.

After ROC analysis, the area under the curve of CTOs and CCCs detected by AIP was 0.596 (95% CI: 0.559–0.633, *p* < 0.001) and 0.597 (95% CI: 0.535–0.658, *p* = 0.002), respectively. The optimal cut-off point for CTOs was 0.28 (Youden index 0.153), with 40.6% sensitivity and 74.7% specificity (Fig. 2), while the optimal cut-off point for CCC poor formation was 0.12, with 92.2% sensitivity and 19.4% specificity (Fig. 3).

### 3.6 Evaluate the Predicted Incremental Value of AIP for CTOs and CCC Poor Formation

**Fig. 4.**
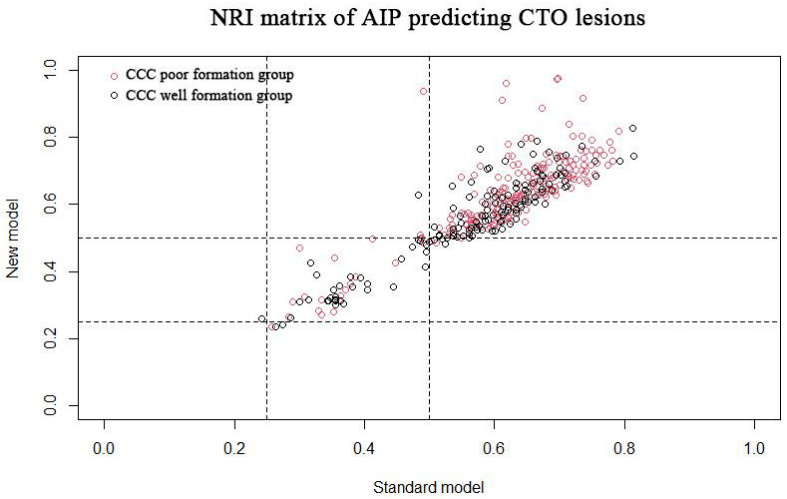
**NRI matrix of AIP predicting CTO lesions.** NRI, net reclassification index; AIP, atherogenic index of plasma; CTO, chronic total occlusive; CCC, coronary collateral circulation.

**Fig. 5.**
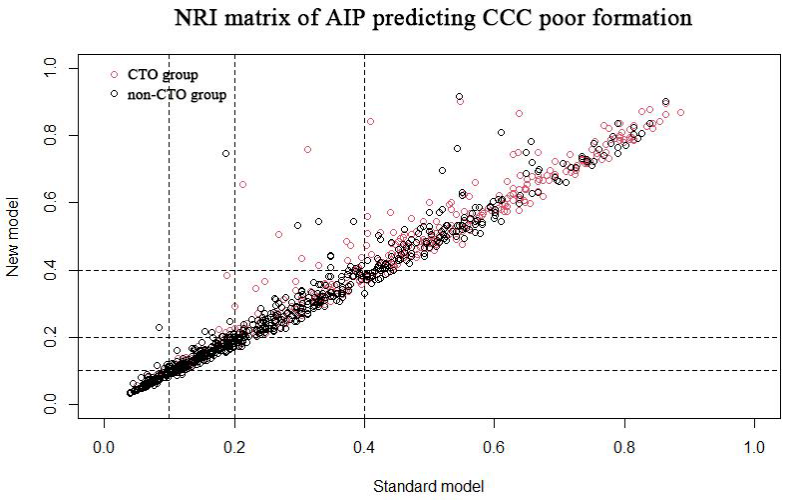
**NRI matrix of AIP predicting CCC poor formation.** NRI, net reclassification index; AIP, atherogenic index of plasma; CCC, coronary collateral circulation; CTO, chronic total occlusion.

We calculated the net reclassification index (NRI) and integrated discrimination index (IDI) to evaluate the predicted increment of AIP on CTOs and CCC poor formation. We constructed a standard model using the risk factors in the above analysis that may be associated with CTOs and CCC poor formation (i.e., age, gender, smoking, diabetes, hypertension for CTOs, and for CCC poor formation, multivessel coronary disease is added). Adding AIP to the standard model forms a new model. Based on previous research [11, 12, 13, 14, 15, 16, 17, 18, 19], we set the cut-off point for CTOs as 0.2, 0.4, 0.8 and the cut-off point for CCC poor formation was 0.25 and 0.5. The final results suggest that the NRI for predicting CTO lesions and poor CCC formation are 0.066 (95% CI: –0.002 to 0.122, *p* = 0.044) and 0.016 (95% CI: –0.039 to 0.183, *p* = 0.761), respectively. The NRI of CTO patients and non-CTO patients were 0.032 (95% CI: –0.018 to 0.054, *p* = 0.083) and 0.034 (95% CI: –0.006 to 0.093, *p* = 0.169), respectively. The NRI of poor and well-formed CCC groups were –0.005 (95% CI: –0.091 to 0.020, *p* = 0.866) and 0.022 (95% CI: –0.017 to 0.224, *p* = 0.740), respectively. The NRI matrix of AIP predicting CTO lesions and poor CCC formation is shown in Figs. 4,5, respectively.

## 4. Discussion

The present study demonstrated that (1) AIP was significantly higher in the CTOs group compared with the non-CTOs group, (2) AIP of CTOs patients with the poor CCC formation group was significantly higher than that of the good CCC formation group, (3) the AIP was positively associated with a higher prevalence of CTOs and CCC poor formation and (4) AIP is a moderate predictor of CTO and poor CCC formation. According to the above results, AIP may provide some assistance in the diagnosis, prognosis, and risk assessment of CTO patients. AIP may also provide some value for judging the poor formation of CCC in CTO patients. The area under the ROC of AIP detection of CTO patients and poor CCC formation is less than 0.7, which means that AIP is only a moderate indicator of CTO and poor CCC formation. This differs from previous findings, possibly due to the characteristics of observational studies and the relatively small sample size of this study.

CTO is one of the leading causes of death in CAD patients because of its complexity and operating procedure difficulty. A well-developed CCC could have a favorable impact on outcomes and functions for CTOs patients. Studies have shown that CCC formation could play an important role in reducing mortality, recurrence of myocardial infarction, MACE and improving the overall prognosis of patients [20, 21, 22]. While CCC formation is involved in disorders of metabolism, evidence has shown that CCC formation is associated with diabetes, metabolic syndrome, and dyslipidemia. The underlying factors contributing to this association may involve endothelial and smooth muscle cell dysfunction, as well as inflammatory cells and cytokines caused by the hostile metabolic environment [3, 23].

Dyslipidemia is traditionally a contributing factor to CAD. Current studies revealed that elevated plasma levels of TG and low plasma concentrations of HDL-C are closely associated with a high risk of CAD, even at or below recommended LDL-C goals [24, 25]. Experimental evidence showed that TG and HDL-C play a role in the pathophysiology of atherothrombosis. TG may provoke atherogenesis by upregulating the production of pro-inflammatory cytokines and enhancing inflammatory response and cell activation [26, 27]. In addition, the elevation of TG could stimulate the secretion of tissue factors from endothelial cells and monocytes, and are related to an increase of fibrinogen and coagulation factors in the plasma [28, 29]. A study suggested that TG could directly contribute to plaque formation and progression [30]. The functionality of HDL is relevant to atheroprotective effects, including anti-inflammatory and anti-thrombotic activities, anti-apoptosis of endothelial cells, and anti-oxidative stress in the process of atherosclerosis [31]. HDL facilitates cholesterol efflux from lipid-rich macrophages within atherosclerotic plaques in the arterial walls. This efflux is mediated by the interactions of apolipoprotein A-I (apoA-I) with adenosine triphosphate-binding cassette transporter A1 (ABCA1). The cholesterol is then transported to the liver for metabolism or biliary excretion [32]. Furthermore, HDL has anti-inflammatory and anti-oxidative effects by regulating endothelial homeostasis and anti-thrombus through attenuation of platelet aggregation and adhesion responses [33]. Thus, it seems more reasonable to use AIP to predict CTOs and CCC formation, since this index represents the ratio of atherosclerosis particles to anti-atherosclerosis particles in the plasma. These protective mechanisms could be related to endothelial function and inflammation in the atherosclerosis process.

The AIP is determined by the logarithmic transformation of the ratio of triglyceride (TG) and high-density lipoprotein cholesterol (HDL-C). Many studies have focused on the relationship between AIP and CAD risk. Previous research links AIP to obesity, hypertension, diabetes, and metabolic syndrome [34]. A study also found that AIP was significantly higher in CAD patients compared with healthy individuals [35], and that AIP was associated with the severity of acute coronary syndrome in young adults [36]. In another study [6], AIP was an independent risk factor for CAD and a higher SYNTAX score ≥23 (OR = 1.623, 95% CI: 1.118–2.358, *p* < 0.01). When the AIP is 2.23, patients may have a higher risk of severe coronary atherosclerosis. Guelker *et al*. [7] found that AIP could predict the complexity of percutaneous coronary intervention (PCI) of CTOs. A higher AIP correlated with a higher J-CTO score, representing the complexity of CTO lesions. AIP was positively related to longer occlusions, longer stent coverage, and a higher number of implanted stents in CTO patients [7]. In this study, AIP was significantly elevated in the CTOs group compared to the non-CTOs group. This result is consistent with another study [2], which also found AIP was positively correlated with the Thrombolysis in Myocardial Infarction (TIMI) score and the Gensini score, when AIP was 0.345, the specificity of the AIP to diagnose CTO was 78.2% and the sensitivity was 65.6%, and that AIP is independently correlated with CTO (OR = 7.024, 95% CI: 5.268–9.365). While the present study showed that AIP was negatively correlated with the Rentrop grade, and that AIP could not only predict the CTOs, with 74.7% specificity, but also predict the CCC formation in CTOs, with a 92.2% sensitivity. The result also showed that AIP was an independent risk factor for CTOs (OR = 3.199, 95% CI: 1.911–5.357) and poor CCC formation (OR = 3.749, 95% CI: 1.628–8.635). However, according to our findings, AIP is only a moderate indicator of CTO and poor CCC formation.

Our study has several limitations. First, all the data in this study were collected from a single hospital and analyzed retrospectively. Therefore, the data could be influenced by measurements, operators, and techniques, and selection bias could exist. Second, we used coronary angiography to evaluate CCC and Rentrop grade, the accuracy of which is far lower than other methods (e.g., CFI). The catheter size and the spatial resolution of the angiography system could have affected the results of the Rentrop grade in this study. Finally, the present study did not completely demonstrate the underlying mechanism between AIP and CCC formation in CTOs patients.

## 5. Conclusions

AIP was significantly higher in the CTOs group and the poor CCC formation group compared with the non-CTOs group and good CCC formation group. We found that AIP was positively correlated with the increased risk of CTOs and poor CCC formation. We found AIP to be a moderate but not powerful indicator for detecting both CTO patients and poor CCC formation. AIP may be used as a noninvasive biomarker to evaluate CTOs and poor CCC formation in clinical practice.

## Data Availability

The datasets used and analyzed during the current study are available from the corresponding author on reasonable request.

## Abbreviations

AIP, atherogenic index of plasma; CAD, coronary artery disease; CTOs, Chronic total occlusions; CCC, coronary collateral circulation; ROC, Receiver operating characteristic; LDL-C, low-density lipoprotein cholesterol; non-HDL-C, nonhigh-density lipoprotein cholesterol; TG, triglyceride; HDL-C, high-density lipoprotein cholesterol; LP(a), Lipoprotein(a); CI, confidence interval; LAD, left anterior descending artery; LCA, left circumflex artery; RCA, right coronary artery; CFI, Collateral Flow Index; MACE, major adverse cardiovascular events; PCI, percutaneous coronary intervention; J-CTO score, Japanese Multicenter CTO Registry score; TIMI, Thrombolysis in Myocardial Infarction.

## Supplementary Material

Supplementary material associated with this article can be found, in the online version, at https://doi.org/10.31083/j.rcm2410305.
