# Supplementary material for: The Atherogenic Index of Plasma is a Predictor for Chronic Total Occlusion and Coronary Collateral Circulation Formation in CTOs Patients
Source: Rev Cardiovasc Med. 2023 Oct 23;24(10):305. doi: 10.31083/j.rcm2410305 (PMC11273106; doi:10.31083/j.rcm2410305)
Supplement: Supplementary file 1 [file 2153-8174-24-10-305-s1.docx]

**Response letter to the editor**

July 7^th^, 2023

Dear editors and reviewers,

We would like to thank the editors and reviewers’ work devoted to our manuscript and we are very grateful for their valuable comments. We have carefully considered all comments from the reviewers and revised our manuscript accordingly. In the following section, we summarize our responses to each comment from the reviewers, which are highlighted in italic. We believe that our responses have well addressed all concerns from the editor and reviewers. We hope our revised manuscript can be accepted for publication.

Revisions are indicated within the text using tracked changes. These revisions were performed to address the reviewers’ concerns. Thank you for allowing us to resubmit our manuscript for your consideration.

**Editor**

**Comment 1.** We have carried out a preliminary layout of your manuscript so that the reviewers could better review your manuscript. Please find the typeset version attached and make revisions on it. (Please download the typeset version in the submission system and make revisions on it.) All changes should be marked or highlighted in the revised manuscript.

***Response 1.*** *We appreciate the editor for taking the time to read our manuscript. We have made revisions in the typeset version and use tracked changes in the manuscript to indicate revisions.*

**Comment 2.** In response to peer-review of your manuscript, it was concluded that the English requires improvement prior to publication. We understand how difficult this can be, especially if English is not your first language. However, for readers to fully understand and benefit from your work, the level of English within manuscripts must be held to a very high standard.

***Response 2.*** *Thanks for the editor's careful inspection.* *We recently completed our English editing services and obtained an English editing certificate meanwhile.*

**Reviewer 1**

**Comment 1.** I would like to thank all authors for this work. My recommendations have been considered, and this work could be published in this current form.

***Response 1.*** *We thank the reviewer for reading our paper carefully and giving the above positive comments.*

**Reviewer 2**

**Comment 1.** The revised manuscript is well-revised and the paper is more interesting to readers. I have just a short comment; the >90% stenosis in the study flow chart should be clearly stated that it is a visual measurement and not a QCA measurement.

***Response 1.*** *We thank the reviewer for this insightful comment. We have made modifications to the flowchart (page 5 Fig. 1). And we also indicate in the legend that we visually assess the degree of coronary artery stenosis during coronary angiography (page 5 line 5).*
